# Correction to: PhaseGen: exact solutions for time-inhomogeneous multivariate coalescent distributions under diverse demographies

**DOI:** 10.1093/genetics/iyaf187

**Published:** 2025-10-07

This is a correction to: Janek Sendrowski, Asger Hobolth, PhaseGen: exact solutions for time-inhomogeneous multivariate coalescent distributions under diverse demographies, *Genetics*, 2025, iyaf135, https://doi.org/10.1093/genetics/iyaf135

In the Funding section of this article, the grant number has been corrected from ‘0069105’ to ‘NNF18OC0031004’.

